# Prevalence of hypertension and its associated factors among adults in Debre Markos Town, Northwest Ethiopia: community based cross-sectional study

**DOI:** 10.1186/s13104-019-4431-9

**Published:** 2019-07-15

**Authors:** Mihretie Kiber, Moges Wube, Habtamu Temesgen, Wubetu Woyraw, Yihalem Abebe Belay

**Affiliations:** 1 Debre Markos Referral Hospital, P.O. Box 269, Debre Markos, Ethiopia; 2 Department of Public Health, College of Health Science, Debre Markos University, P.O. Box 269, Debre Markos, Ethiopia; 3 Department of Human Nutrition and Food Sciences, College of Health Science, Debre Markos University, P.O. Box 269, Debre Markos, Ethiopia

**Keywords:** Hypertension, Prevalence, Debre Markos, Ethiopia

## Abstract

**Objective:**

To assess the prevalence and associated factors of hypertension among adults in Debre Markos town, Northwest Ethiopia. A community based cross sectional study design was employed. Systematic random sampling was used to select 456 study participants. Analysis was performed using SPSS version 20. Binary logistic regression was fitted to show the association between dependent variable and independent variables.

**Result:**

A total of 57 (12.5%) individuals had hypertension in Debre Markos town. Being female [AOR 3.78, 95% CI 1.56, 9.147], consuming animal source fat [AOR 6.28, 95% CI 2.63, 14.99], family history of hypertension [AOR 4.88, 95% CI 1.99, 12.015], age greater than 50 years [AOR 3.31, 95% CI 1.00, 10.99], body mass index ≥ 25 kg/m^2^ [AOR 4.70, 95% CI 1.99, 11.06], excess salt consumption [AOR 6.49, 95% CI 2.83,14.89] and alcohol consumers [AOR 3.19, 95% CI 1.13, 8.99] were found to be statistically significant factors associated with hypertension. The prevalence of hypertension in Debre Markos town is still a public health problem. Being female sex, consuming animal source fat, family history of hypertension, excess salt consumption age greater than 50 years and body mass index > 25 kg/m^2^ were significant factors of hypertension. Therefore, health sectors should take actions to tackle these modifiable risk factors.

## Introduction

High blood pressure is defined as a systolic blood pressure at or above 140 mmHg and/or a diastolic blood pressure at or above 90 mmHg. High blood pressure causes the heart to have to work harder to push blood throughout the body [1, 2].

Globally, nearly one billion adults had hypertension in 2000 and this is estimated to increase to 1.56 billion by 2025. The prevalence of hypertension is rapidly increasing in developing countries and is a major leading cause of death and disability [2]. Globally, 13% of premature deaths occur in developing and developed countries due to hypertension. Today mean blood pressure has been high in many African countries and some European countries [3, 4]. In Africa, hypertension (HTN) is the leading cause of heart failure. Globally, hypertension is responsible for more than half of deaths from stroke [5].

Hypertension is a global public health issue currently. Globally, 40% adults were hypertensive with great regional and residence variations. Similarly, 46% adults in Africa were hypertensive [6, 7]. It contributes to the burden of heart disease, stroke, kidney failure and premature mortality and disability. From cardiovascular disorders, complications of hypertension account for 9.4 million deaths worldwide every year. Hypertension is responsible for at least 45% of deaths due to heart disease and 51% of deaths due to stroke. It is mostly detected incidentally when they are admitted to hospitals [8, 9].

There are risk factors that increase the chances of developing high blood pressure. Of which, Smoking, Diabetes, Being obese or overweight, high cholesterol, unhealthy diet and physical inactivity can be controlled. However, history of high blood pressure, ethnicity, age and gender cannot be modified [10].

In Ethiopia, the prevalence of hypertension is increasing and most of the cases do not know whether they are hypertensive or not before they are hospitalized. Even though studies were done in Ethiopia, there is no documented research done in the study area. Therefore the aim of this study was to assess the prevalence and associated factors of hypertension in Debre Markos town.

## Main texts

### Study design, setting and periods

A community based cross sectional study design was conducted at Debre Markos town. Debre Markos town had a total population of 107,129 of which 55,707 were females (52%) and 51,422 were males (48%). It is located 299 km North West from Addis Ababa and had seven administrative units (Kebeles), three health centers and 3 health posts (26). The study was conducted from February up to March, 2018.

### Participants

All adults in Debre Markos town were the source population and permanent resident adults older than 18 years old in three Debre Markos town Kebeles were included while pregnant female adults and bed ridden patients were excluded.

### Sample size determination and procedure

The sample size determination for first objective was based on the prevalence of hypertension among adults (25%) which was taken from the study conducted in Bahir Dar in 2015 [11], with 95% confidence interval and 5% degree of freedom. The sample size was determined by using single population proportion formula and by adding 10% non-response rate and with design effect of 1.5, the final sample size was 477.

A multistage systematic random sampling technique was used to select the required sample households. From seven Kebeles, 3 Kebeles were selected by lottery method. The sample size was allocated proportionally for each selected Kebeles. The households were selected by using systematic random sampling techniques using a K value of 11. The first household was selected by using the lottery method. Finally, if there were two and above study units within the household, the lottery method was used to select the participants.

### Study variables

Hypertension was the dependent variable and it’s defined as a systolic blood pressure at or above 140 mmHg and/or a diastolic blood pressure at or above 90 mm Hg and known hypertensive cases taken from adults’ age ≥ 18 years. Socio-demographic characteristics: Age, sex, educational status, occupational status, income, ethnicity and religion; Behavioral factors: Alcohol consumption, Cigarette, smoking, chewing chat, and physical exercise, drinking coffee, salt consumption, and animal source fat consumption; Other factors: diabetes mellitus, weight, height, body mass index, family history of hypertension were the independent variables that were used to explain the dependent variable.

### Data collection procedures

Data were collected using a pretested and structured interviewer administered questionnaire. The questionnaire was prepared in English and translated to Amharic, then back translate to English to check the consistency. The questionnaire was adapted from a WHO STEPWISE survey for developing countries and from different literatures [12]. Blood pressure (BP) was measured using a manual sphygmomanometer with participants taking rest before measuring. Portable weight and height measuring scales (PRESTIGE measuring instruments) were used to measure the weight and height of adults who were included in the study by following the standard procedures of weight measurements. The weighing scales were checked and adjusted at zero level between each measurement and the instrument was calibrated daily by known object. Height was measured by following the standard procedures. Two BP measurements were taken for elevated pressures to check the variation and it was taken the average measurement. To assure the data quality, training regarding the study objectives and data collection process was given for data collectors and supervisor for 1 days. Additionally, Pretest was conducted in Dejen town by taking 5% of the total sample size and intensive supervision was done by supervisor and principal investigators throughout the data collection period. Participants who drank caffeine, chat chewers and smokers were made stay for 30 min before BP measurement.

### Data processing and analysis

The collected data were entered to Epi Data version 4.2 and exported to SPSS version 25 for analysis. Descriptive statistics like, frequency and SD were calculated. Binary logistic regression was fitted. Both the bi-variable and multivariable logistic regression analyses were performed to assess the association between dependent and independent variables. Independent variables that showed P < 0.2 at 95% CI in the bivariate logistic regression analysis were included in the multivariable analysis. Finally, variables that showed P < 0.05, with 95% CI were considered statistically significant factors for hypertension.

## Results

### Socio-demographic characteristics of the respondents

A total of 456 adult individuals were participated with the response rate of 95.6%. Of the total, 248 (54.4%) were female participants. Most of the respondents were married (76.3%) while 13 (2.9%) of the respondents were having first degree and above educational level. Almost 97% of the respondents were orthodox religion followers and 40.4% were merchants. The mean age of the respondents was 43.69 ± 14 SD. The majority of the study participants were within age greater than 50 years (Table 1).

**Table 1:** Socio-demographic characteristics of adults in Debre Markos town, North West Ethiopia, 2018

Variables	Category	Frequency	%
Age	≤ 30 years	124	27.2
	31–40 years	111	24.3
	41–50 years	91	20.0
	> 50 years	130	71.5
Sex	Male	208	45.6
	Female	248	54.4
Marital status	Single	47	10.3
	Married	348	76.3
	Widowed	29	6.4
	Divorced	32	7.0
Educational status of respondent	Cannot read and write	92	20.2
	Can read and write	110	24.1
	Grade 1–8	150	32.9
	Grade 9–12	38	8.3
	Certificates and above	66	14.5
Religion of respondents	Orthodox	442	96.9
	Muslim	14	3.1
Ethnicity of respondents	Amhara	449	98.5
	Oromo	6	1.3
	Tigray	1	0.2
Occupational status	Employed	127	27.9
	Merchant	184	40.4
	Housewife	52	11.4
	Student	11	2.4
	Others	82	18.0

### Nutritional status of the respondents

From the study participants, 297 (65.1%) were having normal weight while 13.4% have a BMI less than 18.5 kg/m^2^. But 98 (21.5%) of the participants had BMI ≥ 25 kg/m^2^.

### Blood pressure status of the respondents

The overall prevalence of hypertension was 12.5% and 1.5% were known hypertensive cases. The prevalence of hypertension among female was 18%, while 35% of hypertensive adults had a family history of HTN.

### Dietary and behavioral characteristics of the respondents

From 456 study participants, 313 (68.6%) were drink alcohol, 6 (1.3%) were chat chewers, 4 (0.9%) were known diabetics and 34 (7.5%) had a history of hypertension (Table 2).

**Table 2:** Dietary and behavioral characteristics of respondents among adults in Debre Markos town, 2018

Variables	Category	Frequency	%
Drinking alcohols	Yes	313	68.6
	No	143	31.4
Smoking	Yes	2	0.4
	No	454	99.6
Chewing chat	Yes	6	1.3
	No	450	98.7
Doing physical exercise	Yes	11	2.4
	No	445	97.6
Diabetic patient	Yes	4	0.9
	No	452	99.1
Eating red meat	Yes	367	80.5
	No	89	19.5
Consuming animal source fat	Yes	64	14
	No	392	86
Drinking coffee	Yes	140	30.7
	No	316	69.3
Family history of hypertension	Yes	34	7.5
	No	422	92.5
Known hypertensive patients currently	Yes	7	1.5
	No	449	98.5
Excessive salt consumption	Yes	77	16.9
	No	379	83.1

### Factors associated with hypertension

The multivariable analysis showed that those participants who had an age greater than 50 years were 3.31 times more likely to develop hypertension (AOR 3.31, CI 1.00, 10.99), Being female was 3.8 times more likely to develop hypertension (AOR 3.78, CI 1.56, 9.15). Animal source fat consumption were almost nine times more likely to develop hypertension when compared with those who did not consume animal source fats (AOR 6.28, CI 2.63, 14.99). Alcohol consumers were 3.17 times more likely to develop hypertension than the counterparts (AOR 3.17, CI 1.128, 8.995). Additionally overweight, excess salt consumption history and family history of hypertension were also increasing the risk of hypertension (Table 3).

**Table 3:** Factors associated with hypertension among adults in Debre Markos town, North West Ethiopia, 2018

Variable	Blood pressure reading		COR at 95% CI	AOR at 95% CI	P value
	≥ 140/90 mmhg	< 140/90 mmhg			
Sex					
Male	12 (5.8%)	196 (94.2%)	1	1	< 0.01
Female	45 (18.1%)	203 (81.9%)	3.61 (1.86,7.05)	3.783 (1.565, 9.147)	
Age (years)					
≤ 30	7 (5.6%)	117 (94.4%)	1	1	
31–40	11 (9.9%)	100 (90.1%)	1.84 (0.687, 4.921)	1.54 (.392, 6.025)	
41–50	9 (9.9%)	82 (90.1%)	1.834 (0.657, 5.125)	1.85 (0.472, 7.243)	
> 50	30 (23.1)	100 (76.9%)	5.014 (2.11, 11.91)	3.31 (1.00, 10.99)	< 0.05
Alcohol drinking					
Yes	48 (15.3%)	265 (84.7%)	2.7 (1.285,5.662)	3.186 (1.128, 8.995)	< 0.05
No	9 (6.3%)	134 (93.7%)	1	1	
Animal source fat consumption					
Yes	27 (42.2%)	37 (57.8%)	8.8 (4.74,16.37)	6.282 (2.63, 14.99)	< 0.001
No	30 (7.7%)	362 (92.3%)	1	1	
Family history of HTN					
Yes	26 (35.1%)	48 (64.9%)	6.13 (3.34,11.20)	4.88 (1.985, 12.015)	< 0.01
No	31 (8.1%)	351 (91.9%)	1	1	
BMI					
< 25	23 (6.4%)	335 (93.6%)	1	1	
≥ 25	34 (34.7%)	64 (65.3%)	7.73 (4.28,13.99)	4.70 (1.99,11.065)	< 0.001
Excess salt consumption					
Yes	35 (45.5%)	42 (54.5%)	13.52 (7.26,25.18)	6.492 (2.83,14.896)	< 0.001
No	22 (5.8%)	357 (94.2%)	1	1	

## Discussion

This study showed that the prevalence of hypertension among adults was 12.5%, which was in line with a study conducted in Kenya, Nairobi (13%), southern Ethiopia (13.2%) [13]. But it was lower than the study done in Gondar (28.3%), Addis Ababa, Ethiopia (30%), Jimma, Ethiopia (21.3%), Bahirdar, Ethiopia (25%), Uganda (26.4%), Zambia (32.3%) and Kenya (22.8%) [9, 11, 13–16]. This variation might be due to the difference in the study area, study population and socio demographic status of the study participants.

The odds of developing hypertension among females were four times more likely than when compared with those who were males. This might be due to females are more prone to accumulation of fat tissue than males because females have fat mass than lean mass [17, 18]. This was different from the study conducted in Northwest Ethiopia and southern Ethiopia, which males were a higher at risk than females.

The odds of having hypertension among who consumed animal fat source foods was six times more likely than who did not consume animal source fat. This was due to the fact that those foods which contain animal fat source foods have saturated fats, which leads excess accumulation of fat in the blood vessels, and it leads to atherosclerosis. Additionally, saturated fat obtained commonly found from animals, is the risk of cardiovascular disorders [19].

The odds of having hypertension among older age (age greater than 50) were three times more likely to be hypertensive. This study was in line with the study conducted at North West Ethiopia and Addis Ababa [11, 18, 20, 21]. This might be due to relatively older age could be probably affected by non-communicable diseases including hypertension.

The odds of developing hypertension among those who consumed excessive salt was six times more likely when compared to counterparts. This was in line with the study conducted in southern Ethiopia and northwest Ethiopia [11, 18]. This is due to the fact that, salt increases the risk of hypertension and fluid retention [22].

The odds of developing hypertension among those who had a family history of hypertension were five times more likely when compared with counterparts. This was supported by the study conducted in different parts of Ethiopia [8, 9, 18]. Additionally, the odds of developing hypertension among those who were overweight was almost five times more risk than counterparts. This finding was in line with the studies conducted in Southern Ethiopia [8, 18, 23, 24]. This occurs due to the fact that as BMI levels increases the risk of chronic diseases increases. Increased level of BMI indicates the accumulation of fat tissue.

The odds of developing hypertension among those who drank alcohol was three times prone to hypertension than their counterparts. This finding was supported by other research findings alcohol consumption is a risk factor for hypertension [20, 25, 26]. This might be due to alcohol consumption is associated with cardiovascular diseases including hypertension.

This study revealed that hypertension is the public problem in Debre Markos town. Hypertension was significantly associated with sex, consumption of animal source fat, excess salt utilization, family history of hypertension, age, alcohol consumption, and overweight. Therefore, health care managers and health care providers should focus on the above modifiable risk factors to reduce the prevalence of hypertension.

## Limitation of the study

Inability of performing biochemical tests was the limitation of this study. Due to cross sectional nature of the study, difficult to see the real cause effect relationship. Additionally, there might be the presence of misclassification bias due to self-report of some variables.

## Data Availability

The datasets used and/or analyzed during the current study are available from the corresponding author. The data will not be shared in order to preserve participant anonymity.

## Abbreviations

AOR: adjusted odds ratio
CI: confidence interval
OR: crude odds ratio
BMI: body mass index
BP: blood pressure
BSC: Bachelor of Science
DBP: diastolic blood pressure
HBP: high blood pressure
HTN: hypertension
OPD: outpatient department
SBP: systolic blood pressure
OR: odds ratio
WHO: World Health Organization
